# Tuberculose ostéoarticulaire (mal de Pott exclu): à propos de 120 cas à Abidjan

**DOI:** 10.11604/pamj.2015.21.279.6115

**Published:** 2015-08-12

**Authors:** Mariam Gbané-Koné, Samba Koné, Boubacar Ouali, Kouassi Jean -Mermoz Djaha, Ekoya Ondzala Akoli, Ingrid Nseng Nseng, Edmond Eti, Jean Claude Daboiko, Stanislas André Touré, N'zué Marcel Kouakou

**Affiliations:** 1 Service de Rhumatologie CHU Cocody, Abidjan, Cote d'Ivoire; 2 Service de Traumatologie -Orthopédie CHU Cocody, Abidjan, Cote d'Ivoire; 3 Service de Rhumatologie CHU, Bouaké, Cote d'Ivoire

**Keywords:** Tuberculose, arthrite, ostéite, Tuberculosis, arthritis, osteitis

## Abstract

**Introduction:**

La tuberculose ostéoarticulaire (TOA) représente 2 à 5% de l'ensemble des tuberculoses. Elle demeure d'actualité surtout dans les pays à forte endémicité tuberculeuse. L'objectif était de déterminer la prévalence, les aspects topographiques, radiologiques de la TOA en milieu hospitalier ivoirien.

**Méthodes:**

Les auteurs rapportent une expérience de 11 ans, à travers une étude rétrospective de 120 dossiers de patients atteints de la tuberculose ostéoarticulaire (le mal de Pott est exclu de cette étude). N'ont pas été inclus dans l’étude les dossiers ne comportant pas d'imagerie.

**Résultats:**

L'atteinte extra vertébrale représentait 09,2% de la tuberculose ostéoarticulaire. Il s'agissait de 54 hommes et 66 femmes, l’âge moyen était de 43,13 ans. On notait 123 cas d'ostéoarthrites, et 8 cas d'ostéites des os plats. L'atteinte des membres inférieurs prédominait dans 91,87% des cas. La hanche était la première localisation (45,04%), suivie du genou (25,19%). Les atteintes étaient multifocales dans 20% des cas. L'atteinte osseuse était associée à une tuberculose pulmonaire dans 05,83% des cas. Des localisations inhabituelles ont été rapportées: poignet (n = 2), branches ischiopubiennes (n = 4), atteinte sternoclaviculaire (n = 4), médiopieds (n = 2). Les lésions radiologiques étaient avancées (stades III et IV) dans 55,73% des cas. A la TDM, la prévalence des abcès était de 77%. Un geste chirurgical a été réalisé sur 16 articulations (2 épaules, 13 genoux, une cheville).

**Conclusion:**

La TOA des membres est peu fréquente contrairement à l'atteinte vertébrale. La hanche est la principale localisation. Le retard au diagnostic explique l’étendue des lésions anatomoradiologiques.

## Introduction

Selon l'OMS, la tuberculose (TBC) reste un problème de santé publique majeur. Ces dernières années, on assiste à une recrudescence de la TBC dans les pays développés [1]; pour l'année 2012, ce sont 8,6 millions de personnes qui ont contracté cette maladie dans le monde avec 1,3 million de décès (OMS 2013). Les facteurs de risque sont: la pandémie VIH, les conditions de vie précaire, la migration et le brassage de population [1]. La tuberculose ostéoarticulaire (TOA) représente 2 à 5% de l'ensemble des tuberculoses et 9 à 20% des tuberculoses extrapulmonaires (TBE) [1, 2]. Elle constitue une urgence à l’échelle planétaire (OMS); aussi une meilleure description épidémio-clinique de la TOA permettra un diagnostic précoce et une prise en charge adéquate. Cette étude a été entreprise en vue de déterminer la prévalence, les aspects topographiques, radiologiques de la TOA en milieu hospitalier ivoirien.

## Méthodes

Il s'agissait d'une étude rétrospective sur dossiers de patients hospitalisés pour TOA de Janvier 2005 à Décembre 2014 menée dans le service de rhumatologie au CHU de Cocody. Le diagnostic de TOA a été basé (quand cela a été possible) sur des preuves bactériologiques et ou histologiques. Dans la majorité des cas c’était sur un faisceau d'arguments épidémio- anamnestiques, cliniques, radiologiques et thérapeutiques (une évolution satisfaisante sous traitement antituberculeux). Les patients n'ayant pas d'imagerie n'ont pas été inclus dans l’étude. Les paramètres sociodémographiques, cliniques et radiologiques ont été étudiés.

## Résultats

**Figure 1:**
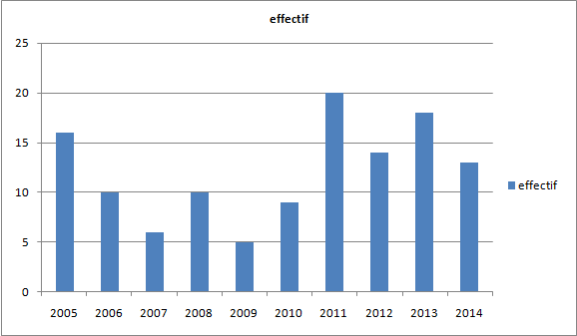
Répartition selon le nombre de cas de TOA extravertébrale par année

**Figure 2:**
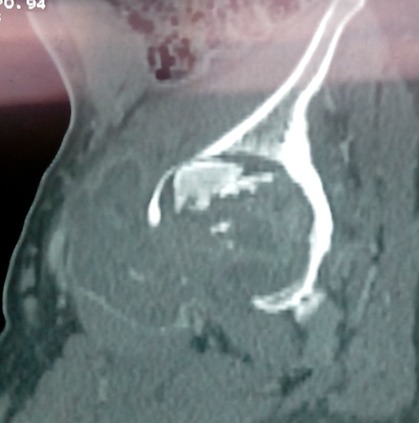
TDM d'une ostéoarthrite tuberculeuse de la hanche avec des abcès

**Prévalence:** la TOA extra vertébrale représentait 02,64% des affections rhumatologiques (120 cas sur 4531 patients hospitalisés) et 09,2% de la TOA. En moyenne, on notait 10,8 cas de TOA extravertébrale par an (Figure 1). **Sexe et âge:** On notait 54 hommes (45%) et 66 femmes (55%), le sex ratio était 0,82. La moyenne d’âge était de 43,13 ans avec des extrêmes allant de 14 à 88 ans.**Antécédents:** une notion de contage tuberculeux avéré a été retrouvée chez 26,7% des patients. Un antécédent de tuberculose chez 06 patients. La sérologie rétrovirale était positive dans 14% des cas. **Clinique:** Les symptômes évoluaient depuis plus de 3 mois dans 90,3% des cas, avec des extrêmes allant de 2 semaines à 2 ans. La douleur était retrouvée chez tous les patients. Elle était inflammatoire dans 81,1% des cas, avec une impotence fonctionnelle dans 18,3% des cas. L'atteinte était unilatérale dans 94,17% des cas. On notait une tuméfaction de l'articulation ou du membre dans 40% et une raideur articulaire dans 67,2% des cas. Une fistulisation spontanée a été notée dans 07 cas. Les signes généraux étaient l'amaigrissement (81,30%), la fièvre (76,70%); l'asthénie (52,20%); les sueurs nocturnes (44,70%). **Topographie:** Sur un total de 131 localisations ostéoarticulaires colligées, on notait 123 cas d'ostéoarthrites (93,90%), et 08 cas d'ostéites des os plats (branches pubiennes et des ailes iliaques). La topographie de ces ostéarthrites est résumée dans le Tableau 1. L'atteinte des membres inférieurs prédominait (n = 113) soit 91,87% des cas. La hanche (Figure 2), était la première localisation (45,04%), suivie du genou (25,19%) et des sacro-iliaques (12,1%). L'atteinte des membres supérieurs représentait 08,13% des cas. Les atteintes étaient multifocales dans 20% des cas. L'atteinte osseuse était associée à une atteinte pulmonaire dans 07cas (05,83%) et une atteinte ganglionnaire dans 05 cas. Les ostéites représentaient 08 cas (4 branches ischiopubiennes, 4 ailes iliaques), elles étaient isolées respectivement dans 1 et 2 cas.

**Tableau 1:** Répartition des atteintes ostéoarticulaires selon la topographie

Site	Effectif	Pourcentage
Hanche	59	45,04
Genou	33	25,19
Sacroiliaque	16	12,21
Cheville	3	02,29
Médiopieds	2	01,53
Epaule	4	03,05
Poignet	2	01,53
Sternoclavicule	4	03,05

**Imagerie Radiographie standard:** Le taux de réalisation de la radiographie standard était de 100%. La radiographie standard paraissait normale ou dégénérative dans 16,66% des cas. L'aspect radiologique des 123 cas d'ostéoarthrites recensés est représenté dans le Tableau 2 . **TDM:** le taux de réalisation de la TDM était de 83,33%, la lésion anatomoradiologique la plus fréquente était une ostéoarthrite (95%). La prévalence des abcès périarticulaires était de 77%. Ces abcès contenaient des calcifications ou séquestres dans 21 cas.**IRM:** le taux de réalisation de l'IRM était de 05%, elle a confirmé l'ostéoarthrite dans tous les cas. **Biologie non spécifique:** le taux de réalisation de la VS et de la CRP était de 100%. On notait un syndrome inflammatoire dans 89,30% des cas, la VS était accélérée dans 69,09% des cas et la CRP était positive dans 76,54%. Le taux de réalisation de l'IDR était de 33,33%. Elle était positive dans 06 cas.**Examen de certitude:** quatre drainages scannoguidés des abcès de la hanche, ont été réalisés. La recherche du BK sur le liquide de ponction était positive dans 7 cas (2 hanches, 5 genoux). La PCR a été réalisée sur les abcès dans 04 cas, avec 02 cas de positivité. **Bilan d'extension:** Chez 7 patients, le BK a été isolé sur les crachats ou le liquide de tubage gastrique. La biopsie ganglionnaire a été réalisée chez 5 patients, elle était positive dans 4 cas. Un geste chirurgical (drainage des abcès, séquestromie, prélèvements biopsiques) a été réalisé sur 16 articulations (2 épaules, 13 genoux et une cheville). L’étude anatomopathologique des prélèvements osseux, a mis en évidence les lésions caséeuses dans 06 cas, des lésions inflammatoires non spécifiques dans 09 cas **Traitement:** Tous les patients ont reçu un traitement antituberculeux d'une durée minimale de 12 mois. Le traitement orthopédique a été réalisé chez 65 patients (attelle ou appareil plâtré). Le traitement chirurgical (drainage des abcès, séquestromie) précoce dans 16 cas.

**Tableau 2:** Répartition des ostéoarthrites selon les stades radiologiques

Stades	Effectif
Stade I	**11**
Stade II	**39**
Stade III	**53**
Stade IV	**20**

## Discussion

La tuberculose vertébrale reste la principale localisation de la TOA [1, 2]. La TOA extravertébrale est rare (1 -5% des cas) [1, 2], dans notre étude elle représentait 09,8% de la TOA, sa fréquence hospitalière était de 02,64% des affections rhumatologiques vues en hospitalisation. La TOA peut toucher les deux sexes et tous les âges [1, 3]. On note une légère prédominance féminine dans notre étude. Dans les zones endémiques, la TOA touche surtout les adultes jeunes [1, 2, 4] alors que dans des pays développés, il a été observé une différence d’âge entre les patients selon leur origine, en effet les immigrants atteints de TOA sont des adultes jeunes, contrairement aux sujets natifs du pays qui sont des personnes âgées [1, 5]. Les facteurs favorisant la tuberculose sont connus; dans les zones endémiques, ce sont surtout les conditions socioéconomiques précaires (malnutrition, mauvaises conditions sanitaires, promiscuité), mais aussi les états de déficit immunitaire (diabète, VIH, corticothérapie au long cours) [1, 2]. Cliniquement, l'arthrite tuberculeuse est une atteinte subaiguë ou chronique, évoluant vers l'aggravation progressive avec une destruction de l'articulation et l'ankylose [1, 2, 6]. Les symptômes cliniques sont peu spécifiques, ce sont: la douleur, le gonflement, la raideur articulaire et l'impotence fonctionnelle [1, 2, 6]; une fistulisation à la peau est possible, de même que la palpation d'abcès froids [6]. L'amyotrophie des muscles péri articulaires est souvent précoce [2]. Par ailleurs des traumatismes mineurs peuvent masquer la lésion sous-jacente et retarder le diagnostic [6]. L'atteinte ostéo articulaire peut être isolée et primitive, l'arthrite tuberculeuse est généralement mono articulaire; cependant 3 à 20% des formes multifocales sont rapportées [1, 2, 6]. L'atteinte multifocale était de 20% dans notre étude, un patient avait jusqu’à 6 localisations (une atteinte pulmonaire associée à 5 localisations osseuses inhabituelles: poignet, cheville, médiopieds, branches ischiopubiennes, épaule). L'atteinte articulaire à Mycobacterium tuberculosis peut se faire soit par voie directe hématogène avec un envahissement de la membrane synoviale, soit par voie indirecte par extension d'un foyer osseux adjacent (mécanisme le plus fréquent) [6]. L′installation insidieuse des symptômes, explique les longs délais de consultation mais aussi la difficulté habituelle du diagnostic [1, 2, 6]. La majorité de nos patients a consulté après un délai de 03 mois. Le retard au diagnostic est unanime pour les auteurs [1, 2, 6, 7], il est à l'origine de l’étendue des lésions radiologiques et de leur caractère destructeur.

La localisation aux membres inférieurs demeure la principale atteinte de la TOA dans notre étude (91,87% des cas), en effet dans une étude similaire réalisée dans le service par Daboiko [8] en 2005, on retrouvait cette prédominance. Dans la littérature, les arthrites tuberculeuses prédominent aux membres inférieurs qui sont atteints dans 60 à 80% des cas [1, 2, 5, 9, 10]. La hanche était la localisation la plus fréquente suivie du genou, idem pour Daboiko [8]. Cette répartition est également retrouvée chez les enfants, en effet dans une étude réalisée par Teklali [7] sur la TOA chez l'enfant, la hanche était l'atteinte principale suivie du genou. La hanche reste la principale localisation dans certains pays en voie de développement [11, 12] alors que dans d'autres études, le genou est la localisation la plus fréquente chez l'adulte devant la hanche [3, 4, 9, 10]. Dans la majorité des cas, le diagnostic de coxite tuberculeuse est fait à un stade tardif (stade radiologique III ou IV) [1, 2]. En ce qui concerne le genou, le diagnostic est fait le plus souvent avant la destruction articulaire, compte tenu du caractère superficiel de cette articulation [1, 2, 10]. L'atteinte sacroiliaque est moins fréquente, c'est la 3ème localisation de la TOA aux membres inférieurs dans notre étude, idem pour Pertuiset [1], Annabi [2] et Teklali [7]. L'atteinte est le plus souvent unilatérale. La tuberculose de la cheville et du pied représente 6 à 8% des localisations, la cheville est la plus touchée [2]. Les lésions sont généralement détectées à un stade destructeur (stades radiologiques III et IV) [2, 10]. La tuberculose des membres supérieurs représente 7 à 14% de l′ensemble des localisations [2]. Elle est nettement moins fréquente que l'atteinte des membres inférieurs. Dans notre étude, elle représentait 08,13% des cas. L'atteinte de l’épaule est rare, elle constitue 1-10,5% des formes osseuses [6]. Certaines localisations sont très rares (poignet, doigts, coude, sternoclaviculaire [1, 2, 10]. Nous n'avons pas eu de localisations au coude ni aux doigts dans notre étude. Par contre deux atteintes du poignet ont été notées.

L'ostéite tuberculeuse des os plats est rare, contrairement à celle des os longs [10, 13, 14]. Dans notre étude, elle représentait 06,1% des cas. Ces ostéites peuvent siéger au bassin (ischion), aux côtes, au crâne, à l'omoplate, au sternum ou à la clavicule [1]. Nous avons eu 04 cas d'atteinte des branches ischiopubiennes et également 04 cas d'ostéite de l'aile iliaque. Martini [1, 10] a proposé une classification radiologique des lésions tuberculeuses en quatre stades évolutifs, celle-ci est dérivée de celle de David-Chaussé (Tableau 3). Plus de la moitié des lésions (55,73%) était déjà à un stade avancé (stade III et IV). Dans une série marocaine [9], 52% des patients avait également des lésions de stade III ou IV. Le retard au diagnostic pourrait expliquer ces lésions avancées [1, 2]. Le stade radiographique est fonction du stade auquel est porté le diagnostic, les signes apparaissant progressivement [15]. L’évolution terminale peut se faire vers l'ankylose. L'apport de la TDM est indéniable dans le diagnostic de la TOA [1, 2, 7]. Elle permet une analyse fine des lésions osseuses et leur étendue (ostéolyse, ostéosclérose, périostose, séquestre), aussi, elle met en évidence des abcès des parties molles, qui peuvent contenir des calcifications évocatrices [16]. La prévalence des abcès était élevée dans notre étude (77%). Le diagnostic de tuberculose devrait être confirmé par l'isolement de M. tuberculosis soit lors de l'analyse histologique, soit par les cultures bactériologiques ou idéalement par les deux [6, 7, 17, 18]. Cependant la tuberculose osseuse étant une lésion paucibacillaire, il est souvent difficile d'isoler le germe dans les prélèvements [6, 18]. Les prélèvements biopsiques, peuvent être réalisés sous arthroscopie ou par arthrotomie [1, 2, 6, 7]. La biopsie à ciel ouvert est préférable dans les pays en voie de développement où les plateaux techniques sont souvent très limités et ne permettent pas la réalisation courante de gestes radioguidés [6]. Le traitement antituberculeux précoce est très efficace et permet la restitution ad integrum, il est au besoin, associé à un geste de drainage [19]. En plus du traitement médicamenteux, 16 patients ont bénéficié d'une arthrotomie dans notre étude. Une arthrotomie est réalisée pour une biopsie diagnostique, un drainage d'abcès, une synovectomie, une exérèse de séquestres et de fistules chroniques [20]. Dans les arthrites tuberculeuses périphériques, les séquelles fonctionnelles sont favorisées par le retard diagnostique et thérapeutique [21]. Dans ces cas, une chirurgie réparatrice peut alors être secondairement réalisée (chirurgie conservatrice, arthrodèse, prothèse) [20].

**Tableau 3:** Classification radiologique des arthrites tuberculeuses selon la classification de David-Chaussé revisitée par Martini

Stade	Description
Stade I	Synovite pure
	Discrète ostéoporose épiphysaire
Stade II	Atteinte osseuse débutante
	Présence d'une ou de plusieurs géodes ou érosions osseuses juxta-articulaires
	Discret pincement de l'interligne articulaire
Stade III	Atteinte destructrice
	Nombreuses géodes et érosions osseuses
	Important pincement de l'interligne
Stade IV	Importante destruction avec déformation
	Atteinte destructrice complète de l'articulation, avec déformation articulaire.

## Conclusion

La TOA des membres est peu fréquente contrairement à la localisation vertébrale, toutes les articulations peuvent être touchées, cependant l'atteinte des articulations portantes reste prépondérante. Il faut pouvoir évoquer le diagnostic devant toute monoarthrite chronique d’évolution insidieuse et faire une prise en charge précoce afin d’éviter les séquelles fonctionnelles graves. En cas de lésions très importantes, l'arthrotomie a un double intérêt: diagnostique (prélèvements biopsiques) mais aussi thérapeutique (drainage de volumineux abcès, séquestromie).
